# A Single-Source Pilot Study of Machine Learning-Assisted Microwave S-Parameter Screening for Glucose Syrup and Water Adulteration in Grape Molasses

**DOI:** 10.3390/s26165114

**Published:** 2026-08-12

**Authors:** Mustafa Alptekin Engin, Mehmet Cakir, Turan Cakil

**Affiliations:** 1 Department of Electrical and Electronics Engineering, Bayburt University, 69000 Bayburt, Türkiye; maengin@bayburt.edu.tr; 2 Department of Software Development, Kamil Özdağ Faculty of Science, Karamanoğlu Mehmetbey University, 70200 Karaman, Türkiye; turancakil@kmu.edu.tr

**Keywords:** grape molasses, food adulteration, microwave sensing, S-parameters, machine learning, group-blocked validation

## Abstract

Grape molasses, known as pekmez in Turkish, is a traditional concentrated fruit product that may be adulterated with cheaper sweeteners or water. This study evaluated broadband microwave S-parameter measurements as a rapid, non-destructive screening approach for detecting glucose syrup substitution and water dilution in grape molasses. Nine physical mixture groups were prepared, including pure grape molasses, glucose syrup–substituted mixtures at 5–30%, pure glucose syrup as an endpoint reference, and water-diluted mixtures at 10–30%. For each group, five consecutive technical measurements were recorded using a Libre vector network analyzer connected to a WR-229 waveguide-based two-port setup over 3.30–4.90 GHz. All mixtures were prepared from a single commercial grape molasses source and a single glucose syrup source; the results should therefore be interpreted as pilot-scale, single-source evidence rather than a generalizable screening method. Glucose syrup substitution produced a systematic upward shift in S_11_ resonance frequency. In the practical 0–30% range, S_11_ resonance frequency showed a strong linear relationship with glucose syrup content on the calibration data (R^2^ = 0.9955; inverse prediction error = 0.72 percentage points), though this reflects calibration fit rather than independently validated prediction accuracy. Water dilution produced stronger S_21_ attenuation. The detection principle is based on the complementary use of S_11_ resonance behavior for glucose syrup substitution and S_21_ transmission loss for water dilution. In group-blocked point-wise classification, the Ensemble Bagged Trees classifier achieved 88.79% accuracy and a macro-F1 score of 0.874. These results indicate that S_11_ and S_21_ provide complementary proof-of-concept indicators for controlled-mixture screening of grape molasses adulteration.

## 1. Introduction

Grape molasses, also known as pekmez, is a traditional concentrated fruit product that is made by heating clarified grape juice to decrease the amount of water in it [1]. The concentration process results in a denser matrix, with a high content of natural sugars, minerals, organic acids, and phenol compounds. Grape molasses is eaten as is and has also been used as a flavoring for a number of food products due to its flavor and nutritional benefits. It can be different depending on grape cultivar, production process, concentration process and raw material properties [2,3,4].

Due to the high sugar content and its commercial significance, grape molasses can be susceptible to economically motivated adulteration. Adulteration can be the inclusion of cheaper sweeteners like glucose syrup, fructose syrup, sucrose syrup, sugar beet syrup or high fructose corn syrup or water [5,6,7,8]. While conventional physicochemical parameters such as Brix, pH, density and viscosity can show deviations, they may not be adequate in the presence of certain adulterants, which are chosen to simulate the natural composition and texture of grape molasses [6,8,9].

Stable carbon isotope ratio analysis is reported to be more reliable to detect adulteration with syrups of different botanical origins. Tosun was able to identify corn-derived glucose and high fructose corn syrup (HFCS) in mulberry pekmez using isotope ratio analysis [7], and Yücel et al. applied the same technique to carob, grape, fig, and mulberry pekmez [5]. Jamali et al. further analyzed Iranian grape molasses adulterated with glucose, fructose and sugar beet syrups by isotope ratio analysis (IRA) [8]. Isotope-based analysis is very powerful and can be used in the laboratory with the appropriate equipment, personnel, and time; however, it is not easily translatable to the field for quick screening. Spectroscopic methods provide faster alternatives for adulteration screening. Attenuated total reflectance–Fourier transform infrared (ATR-FTIR) spectroscopy combined with chemometric analysis has demonstrated promising performance for pekmez authentication. Yaman and Velioğlu showed that partial least squares-discriminant analysis (PLS-DA) achieved high sensitivity and specificity in distinguishing pure and adulterated pekmez samples, while PLS regression was used to estimate adulteration levels [9]. However, dataset design, measurement conditions, and validation strategies must be carefully controlled to ensure reliable performance across different adulterants, batches, and production sources. Yaman and Velioğlu showed that partial least squares-discriminant analysis (PLS-DA) was very sensitive and specific for distinguishing the pure and adulterated pekmez samples and PLS regression could be used to estimate the adulteration levels [9]. But, the design of the dataset, the measurement conditions and the validation of the spectroscopic calibration models must be carefully controlled to ensure that they can be validated over different types of adulterants, various batches and production sources.

Microwave sensing has gained increasing attention as a rapid, non-destructive, and cost-effective method for food quality assessment [10,11]. The interaction between electromagnetic (EM) waves and food material depends on the dielectric properties of the material, which are influenced by the water content, sugar concentration, ionic composition, viscosity and molecular mobility of the food [12]. For 2-port microwave measurement, S_11_ will give reflection, impedance mismatch and resonance data, while S_21_ will give transmission, attenuation and dielectric loss data. Thus, the evaluation of the two signals, S_11_ and S_21_, can complement in order to capture compositional changes in liquid and semi-liquid foods. Microwave-based sensing has previously been explored in different food authentication and quality-control applications. Naderi-Boldaji et al. used dielectric spectroscopy combined with linear discriminant analysis (LDA) and support vector machine (SVM) to identify adulteration in grape syrup [13]. Hasar et al. developed a simple microwave system based on S_21_ measurements to quantify water adulteration in honey [14]. Microwave dielectric spectroscopy has also been applied to quality and anti-adulteration control of vegetable oils [15]. Recent studies have further combined S-parameter measurements with machine learning (ML) for adulterant identification in extra virgin olive oil and packaged food products [16,17]. Low-cost open-ended coaxial measurement techniques have also been proposed for the characterization of liquid foods in industrial applications [18]. Resonance frequency has also been used as a proxy for online Brix measurement in syrups [19].

ML methods have increasingly been used to interpret complex spectroscopic and sensor-based data in food adulteration detection, because they can identify discriminative patterns that may not be evident from individual spectral variables [20,21,22]. A key methodological issue in spectral machine learning is the risk of data leakage. If individual frequency points from the same physical sample are treated as independent observations and randomly divided into training and test sets, the resulting accuracy can be overestimated. This problem is particularly important in high-dimensional datasets with limited numbers of physical samples. Roberts et al. and Vabalas et al. emphasized the importance of appropriate cross-validation (CV) strategies for structured or small-sample datasets. Therefore, group-blocked validation is more appropriate in the present context, because technical repetitions were first averaged at the mixture-group level and all frequency points belonging to the same mixture group were kept together within the same validation fold [23,24].

Although spectroscopic and dielectric methods have been explored for food adulteration screening, limited information is available on the broadband microwave S-parameter response of grape molasses adulterated with glucose syrup and water. In this study, S_11_ and S_21_ responses were measured for pure grape molasses, grape molasses–glucose syrup mixtures, pure glucose syrup, and grape molasses–water mixtures over the 3.30–4.90 GHz frequency range. The objectives were to identify the electromagnetic signatures of the two adulteration mechanisms, evaluate S_11_ resonance frequency as an indicator of glucose syrup substitution, assess S_21_ transmission loss as an indicator of water addition, and perform leakage-controlled point-wise classification using group-blocked validation. This study does not propose a new VNA architecture, waveguide design, or machine-learning algorithm. Its contribution lies in the application-specific, controlled evaluation of the complementary broadband S_11_ and S_21_ responses of grape molasses adulterated with glucose syrup and water, with particular emphasis on leakage-controlled validation and on distinguishing the electromagnetic signatures of two different adulteration mechanisms. The study specifically examines whether S_11_ resonance behavior and S_21_ transmission loss provide complementary indicators for these two adulteration mechanisms. Therefore, the work is positioned as a proof-of-concept screening study intended to clarify the measurement principle and support future multi-batch validation studies. We explicitly frame this work as a single-source pilot study: all measurements originate from one grape molasses batch and one glucose syrup batch, and the findings should be read as preliminary evidence motivating larger multi-batch investigations rather than as a validated screening method.

## 2. Materials and Methods

### 2.1. Overall Workflow and Analytical Rationale

**Figure 1:**
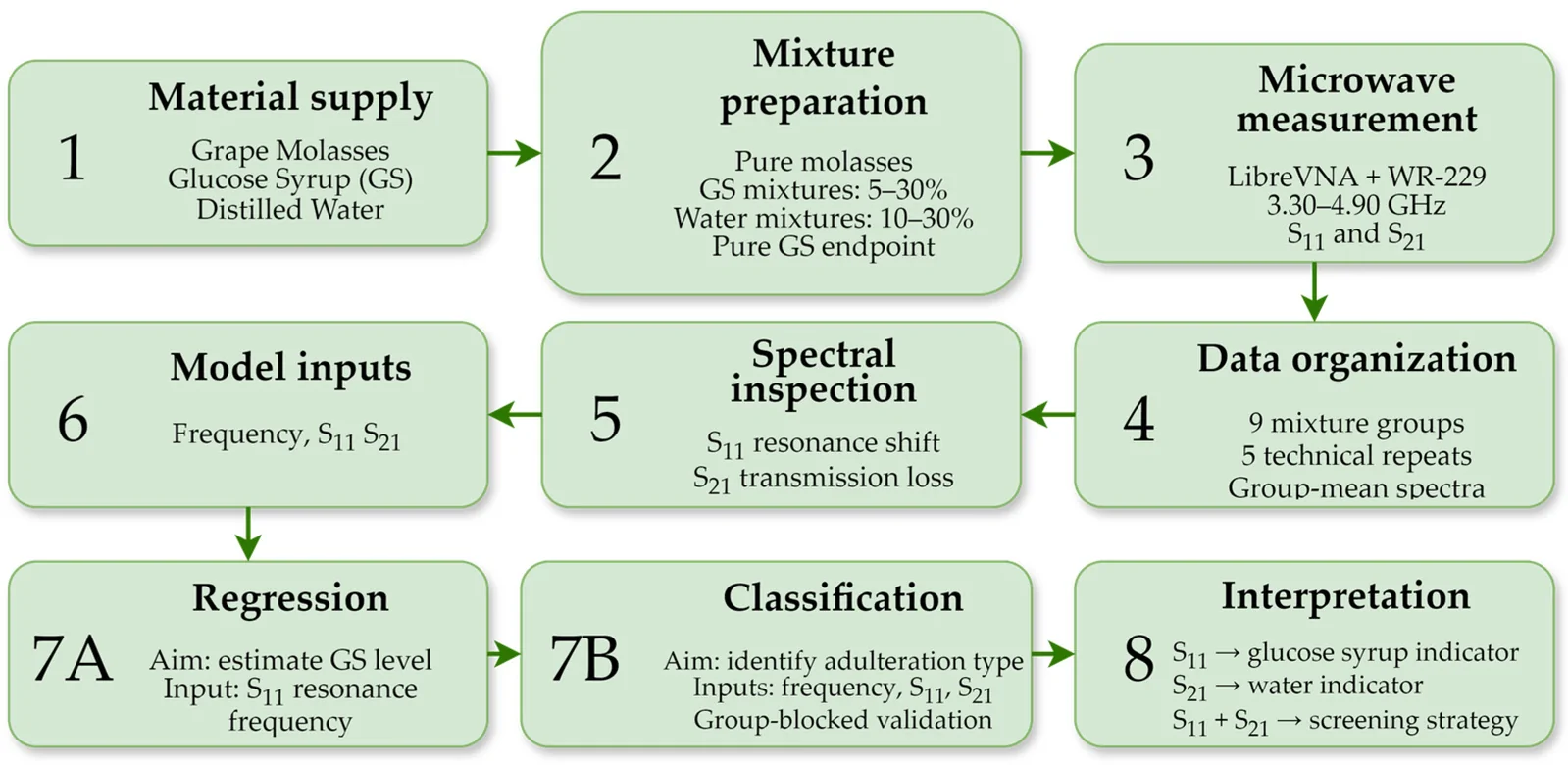
Schematic overview of the proof-of-concept microwave screening approach. The workflow summarizes controlled mixture preparation, S-parameter measurement, spectral inspection, regression, group-blocked classification, and interpretation of S_11_ and S_21_ responses.

The overall methodological workflow of the study is shown in Figure 1. The study was designed to evaluate whether microwave S-parameter measurements could provide physically interpretable indicators for two different adulteration mechanisms in grape molasses: glucose syrup substitution and water dilution. First, controlled mixture groups were prepared using commercial grape molasses, glucose syrup, and distilled water. Second, broadband S_11_ and S_21_ measurements were obtained using a two-port WR-229 waveguide setup. Third, the measured spectra were inspected to identify resonance shifts and transmission-loss patterns. The model inputs were then defined according to the analytical purpose: S_11_ resonance frequency was used for regression, whereas frequency, S_11_, and S_21_ values were used for point-wise classification. Because spectral points from the same physical mixture are structured observations rather than independent samples, group-blocked validation was used for classification to reduce data leakage and avoid over-optimistic performance estimates [23,24].

### 2.2. Materials and Mixture Preparation

Commercial grape molasses (M) was purchased from a local market and used as the authentic reference sample. Commercial food-grade glucose syrup (GS) and distilled water (W) were used as adulterants. All materials were stored at room temperature in sealed containers until sample preparation. All mixtures were prepared on a weight/weight basis (*w*/*w*) using an analytical balance. This approach was preferred to minimize volumetric errors associated with the high viscosity of grape molasses and glucose syrup and the density differences among the components. Four grape molasses–glucose syrup mixtures were prepared at 5%, 10%, 20%, and 30% glucose syrup levels. Pure glucose syrup was also included as an endpoint reference for the glucose syrup series. Three grape molasses–water mixtures were prepared at 10%, 20%, and 30% added water levels. In total, nine mixture groups were evaluated. For example, the 95M5GS sample consisted of 95 g grape molasses and 5 g glucose syrup, whereas the 90M10W sample consisted of 90 g grape molasses and 10 g distilled water. Full definitions are given in Table 1.

The two-mixture series were selected to represent different adulteration mechanisms. Glucose syrup substitution represents the replacement of grape molasses with a sugar-rich adulterant, whereas water addition represents dilution and increased dielectric loss. This design allowed the electromagnetic responses of two different adulteration mechanisms to be compared under controlled mixture conditions.

### 2.3. Microwave S-Parameter Measurement Setup

**Figure 2:**
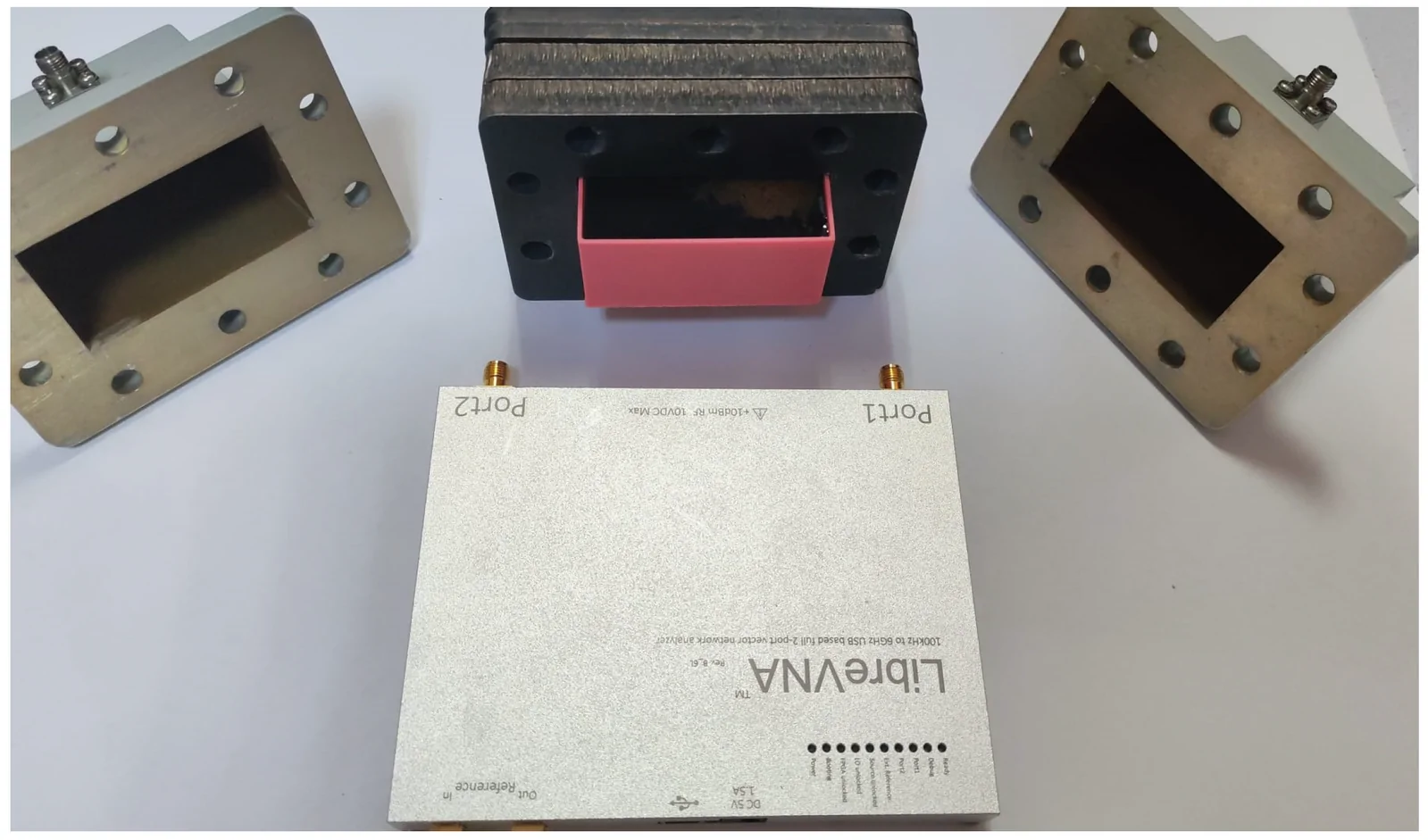
Experimental setup used for microwave S-parameter measurements of grape molasses mixtures, including the LibreVNA, WR-229 waveguide sections, sample container, and 3D-printed holder used for mechanical alignment and repeatable positioning of the sample cell.

The microwave S-parameter measurements were performed using a LibreVNA vector network analyzer (100 kHz–6 GHz, Zhejiang Zhike Technology Co., Ltd., Zhejiang, China) connected to a WR-229 waveguide-based two-port measurement setup (3.30–4.90 GHz, Chengdu AINFO Inc., Chengdu, China), as shown in Figure 2. The liquid sample was placed in a dedicated container/cell located between the two WR-229 waveguide sections. The sample cell dimensions were designed to match the WR-229 waveguide aperture, approximately 58.17 mm × 29.08 mm. For each measurement, 30 mL of liquid sample was placed into the cell, and the same filling condition was maintained for all mixture groups to ensure comparable waveguide loading.

During the measurements, the container was supported by a 3D-printed plastic holder to maintain a fixed and repeatable position between the WR-229 waveguide sections. The plastic holder was used only for mechanical alignment and was not intended to provide electromagnetic shielding. To account for the contribution of the holder and container, an empty-holder reference measurement was recorded before the sample measurements and used for background correction. The corrected spectra were used in the subsequent analysis to emphasize the electromagnetic contribution of the liquid sample.

The reflection coefficient (S_11_) and transmission coefficient (S_21_) were measured over the frequency range 3.30–4.90 GHz with equally spaced frequency points of 1001. The magnitude responses of the reflection coefficient (S_11_) and transmission coefficient (S_21_) were recorded in dB. S_11_ was used to evaluate impedance mismatch and resonance-related behavior in the sample-loaded waveguide section, whereas S_21_ was used to evaluate transmission loss and attenuation associated with dielectric absorption. To minimize the temperature effects on the dielectric response of the samples, measurements were carried out at room temperature (25 ± 1 °C) [25].

The mixtures were gently stirred before each measurement in order to obtain homogeneity of the sample and to minimize any concentration gradients in the viscous matrix. Before the measurement session, a two-port calibration was performed using the calibration kit supplied with the LibreVNA to minimize systematic errors associated with cables, connectors, and port mismatch. All sample groups were then measured under the same experimental conditions. After each sample group, the container/cell and the WR-229 waveguide measurement region were carefully cleaned and dried to prevent cross-contamination between mixtures. For each mixture group, five consecutive technical measurements were performed under the same fixture-loading condition.

### 2.4. Data Structure and Validation Strategy

The dataset consisted of nine physical mixture groups. For each group, five consecutive technical measurements were recorded, resulting in 45 measured spectra and 45,045 spectral data points before averaging. For the analytical modeling steps used in this study, the five repeated spectra of each group were averaged point-by-point, producing nine group-mean spectra with 1001 frequency points each. Therefore, the technical repetitions were used to improve the stability of the group-level spectral response, but they were not treated as independent experimental samples. The independent experimental unit was the physical mixture group, not the individual frequency point. Thus, frequency-wise observations from the same mixture group were considered structured measurements belonging to the same physical group. To reduce data leakage and avoid over-optimistic performance estimates, group-blocked validation was used in the classification analysis. In each validation fold, all frequency points belonging to one mixture group were held out together. The main classification task compared glucose syrup substitution with water dilution. The pure grape molasses group was excluded from this binary task. The glucose syrup class included 95M5GS, 90M10GS, 80M20GS, 70M30GS, and the pure glucose syrup endpoint reference. The water class included 90M10W, 80M20W, and 70M30W. This resulted in 8008 point-wise observations from eight group-mean spectra. The binary task was considered the main classification analysis because it directly tested whether the microwave S-parameter signatures could distinguish between the two adulteration mechanisms.

### 2.5. Spectral Inspection and Model Inputs

The first step in the analysis was the visual inspection of the S_11_ and S_21_ spectra. Group-mean spectra were plotted over the complete frequency range for all mixture groups and separately for the glucose syrup and water series. These plots were used to inspect resonance shifts, attenuation changes, and adulterant-dependent spectral patterns. For regression analysis, the S_11_ resonance frequency was determined from the frequency at which the S_11_ response reached its minimum value in the glucose syrup series. This parameter was selected because the S_11_ resonance frequency showed a systematic shift with increasing glucose syrup concentration. For point-wise classification, each observation corresponded to one frequency point from a group-mean spectrum. Frequency, S_11_, and S_21_ values were used as the only input variables. No additional spectrum-level descriptors or derived features were used as classifier inputs. This approach was preferred to test whether the directly measured microwave responses contained sufficient discriminative information for distinguishing glucose syrup substitution from water dilution.

### 2.6. Regression Analysis

Linear regression was used to evaluate the relationship between S_11_ resonance frequency and glucose syrup content. The purpose of this analysis was to test whether S_11_ resonance frequency could serve as a simple quantitative indicator of glucose syrup substitution under controlled mixture conditions. Two calibration ranges were considered. The first model was fitted over the practical adulteration range of 0–30% glucose syrup, including pure grape molasses as the 0% reference. This range represents the main adulteration interval evaluated in the study. The second model included pure glucose syrup as a 100% endpoint reference to describe the broader endpoint-supported calibration trend. The 100% glucose syrup group was treated as an electromagnetic endpoint rather than a realistic commercial adulteration level. For both models, performance was evaluated using the slope, intercept, coefficient of determination (R^2^), root mean square error in the frequency domain, and inverse prediction error expressed in glucose syrup percentage points. Root mean square error (RMSE) was calculated as the square root of the mean squared residuals between the measured and fitted resonance frequencies. The inverse prediction error was calculated by dividing RMSE in the frequency domain by the regression slope, thereby expressing the frequency-domain error as glucose syrup percentage points.

### 2.7. Classification Analysis

Point-wise classification was used as an exploratory analysis to test whether the combined frequency, S_11_, and S_21_ responses could distinguish glucose syrup substitution from water dilution. The classification results were interpreted as leakage-controlled evidence of separability rather than as final diagnostic performance. The main binary classification task compared the glucose syrup and water adulteration mechanisms. The pure grape molasses group was excluded from this task. The classification dataset contained 8008 frequency-wise observations nested within only eight physical mixture groups. Accordingly, the effective independent experimental sample size for this binary classification task was eight mixture groups, not 8008 observations. The frequency-wise observations were retained only to represent the spectral response within each mixture, and they were not regarded as independent experimental replicates. A leave-one-mixture-group-out validation strategy was used. In each fold, one physical mixture group was held out for testing, while the remaining mixture groups were used for model training. Thus, all frequency points belonging to the same physical mixture group were kept together within the same validation fold. Ten classifiers were evaluated: Logistic Regression, Gaussian Naive Bayes, Linear Support Vector Machine, radial basis function Support Vector Machine, k-nearest neighbors, Decision Tree, Random Forest, Extra Trees, Gradient Boosting, and Ensemble Bagged Trees. Frequency, S_11_, and S_21_ were used as the only input variables. For models sensitive to feature scaling, standardization was applied within the cross-validation pipeline to avoid information leakage from the test group into the training process. For the Ensemble Bagged Trees classifier, an ensemble of 10 bagged decision trees was used. Bootstrap sampling was enabled, and all input features and samples were allowed to be used during ensemble construction. Each base decision tree was configured with the Gini criterion, a maximum depth of 2, a minimum leaf size of 50, and balanced class weights. This configuration was used to limit model complexity while allowing ensemble-based decision boundaries within the group-blocked validation framework.

Model performance was evaluated using accuracy, balanced accuracy, macro-precision, macro-recall, and macro-F1 score. Macro-F1 was considered the primary comparison metric because the numbers of glucose syrup and water mixture groups were not equal.

## 3. Results

The microwave S-parameter responses of pure grape molasses, glucose syrup–substituted mixtures, pure glucose syrup, and water-diluted mixtures were evaluated over the 3.30–4.90 GHz frequency range. The results are presented in three parts: spectral signatures of adulteration mechanisms, regression analysis for glucose syrup estimation, and exploratory classification of glucose syrup versus water adulteration.

### 3.1. Spectral Signatures of Glucose Syrup and Water Adulteration

**Figure 3:**
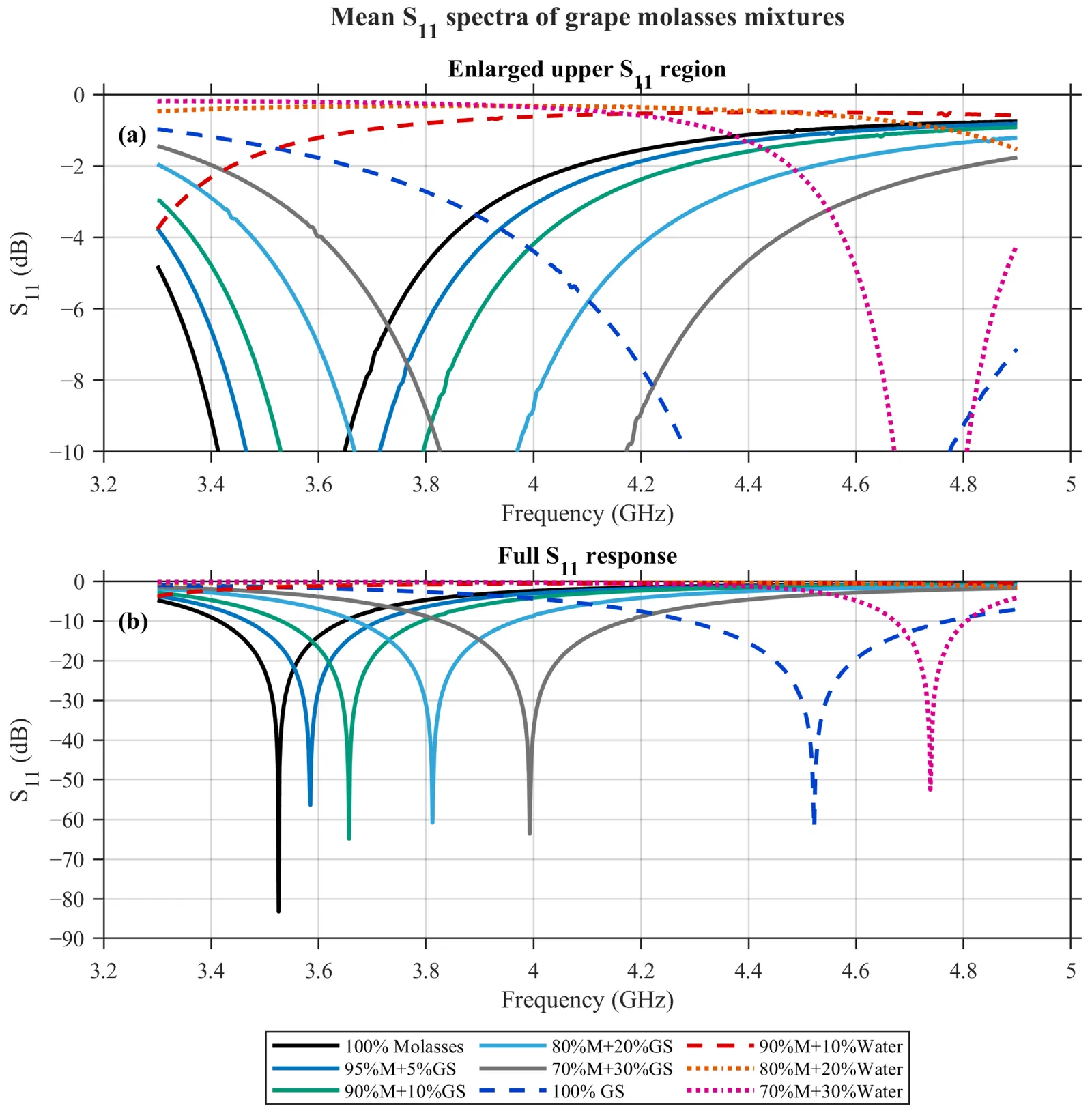
Mean S_11_ spectra of pure grape molasses, glucose syrup–adulterated mixtures, pure glucose syrup, and water-adulterated mixtures over the 3.30–4.90 GHz frequency range: (**a**) enlarged view of the upper S_11_ region (0 to −10 dB); (**b**) full S_11_ response over the complete plotted range (0 to −90 dB). Each curve represents the point-wise mean of five consecutive technical measurements.

**Figure 4:**
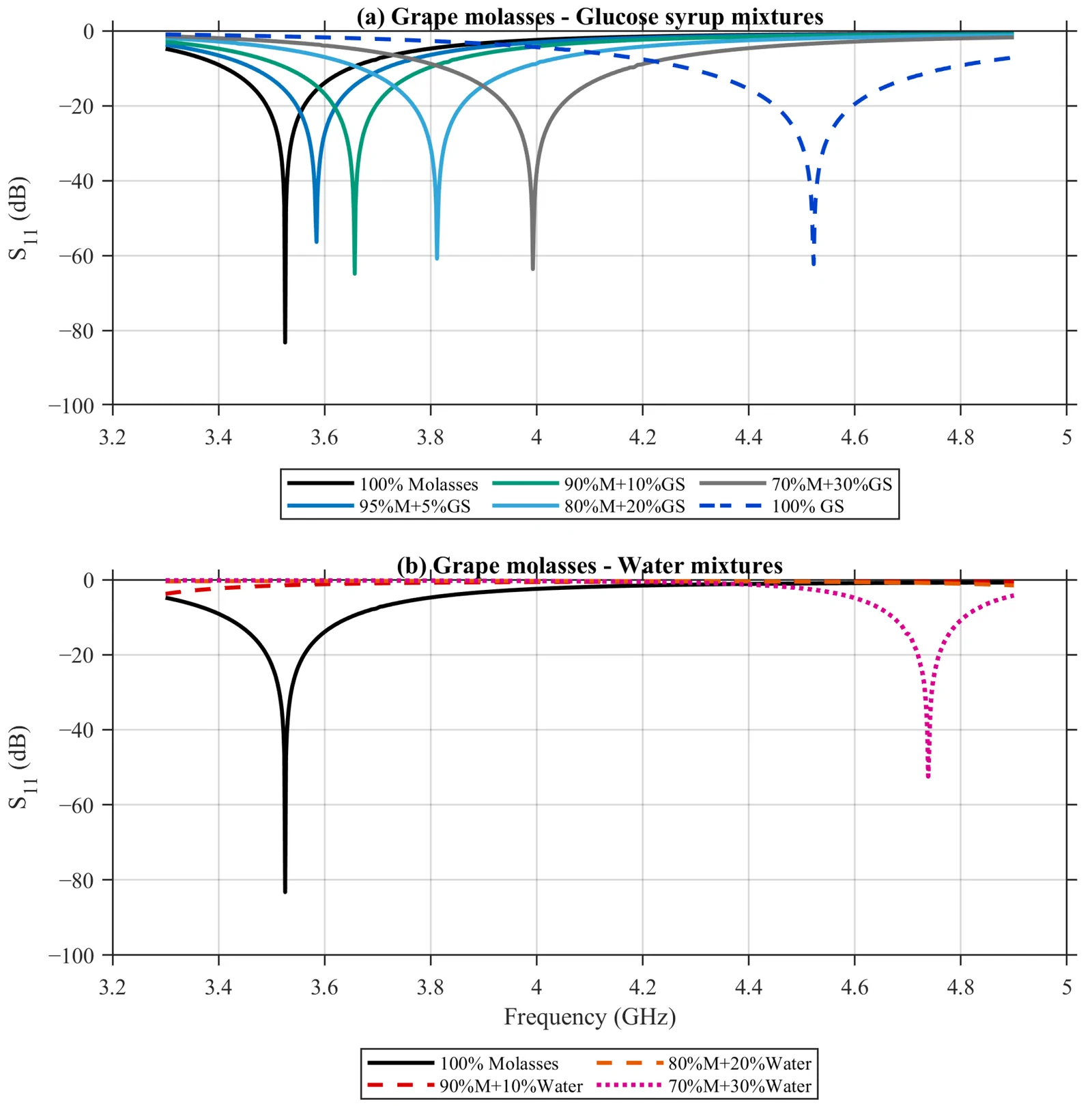
Comparison of mean S_11_ spectral responses of grape molasses mixtures: (**a**) glucose syrup substitution series, including pure grape molasses, 5–30% glucose syrup mixtures, and pure glucose syrup; (**b**) water dilution series, including pure grape molasses and 10–30% water-added mixtures. The glucose syrup series exhibits a progressive shift in the S_11_ resonance frequency, whereas the water-diluted mixtures show a distinctly different resonance response.

The S_11_ spectra showed a clear resonance shift along the glucose syrup series. Pure grape molasses had an S_11_ minimum at 3.5256 GHz, whereas pure glucose syrup had an S_11_ minimum at 4.5224 GHz. The intermediate glucose syrup mixtures showed a monotonic upward shift, with resonance frequencies of 3.5848, 3.6568, 3.8120, and 3.9928 GHz for the 5%, 10%, 20%, and 30% glucose syrup mixtures, respectively. This systematic trend indicates that S_11_ resonance frequency is sensitive to glucose syrup substitution. In contrast, the water-diluted mixtures showed a less regular S_11_ response. The 10% and 20% water mixtures exhibited weak or boundary-dominated resonance behavior, whereas the 30% water mixture showed a resonance-like minimum at 4.7384 GHz. Therefore, the S_11_ resonance frequency was more suitable for monitoring glucose syrup substitution than for quantifying water dilution. The mean S_11_ spectra of all groups are shown in Figure 3, and the glucose syrup and water series are compared separately in Figure 4.

**Figure 5:**
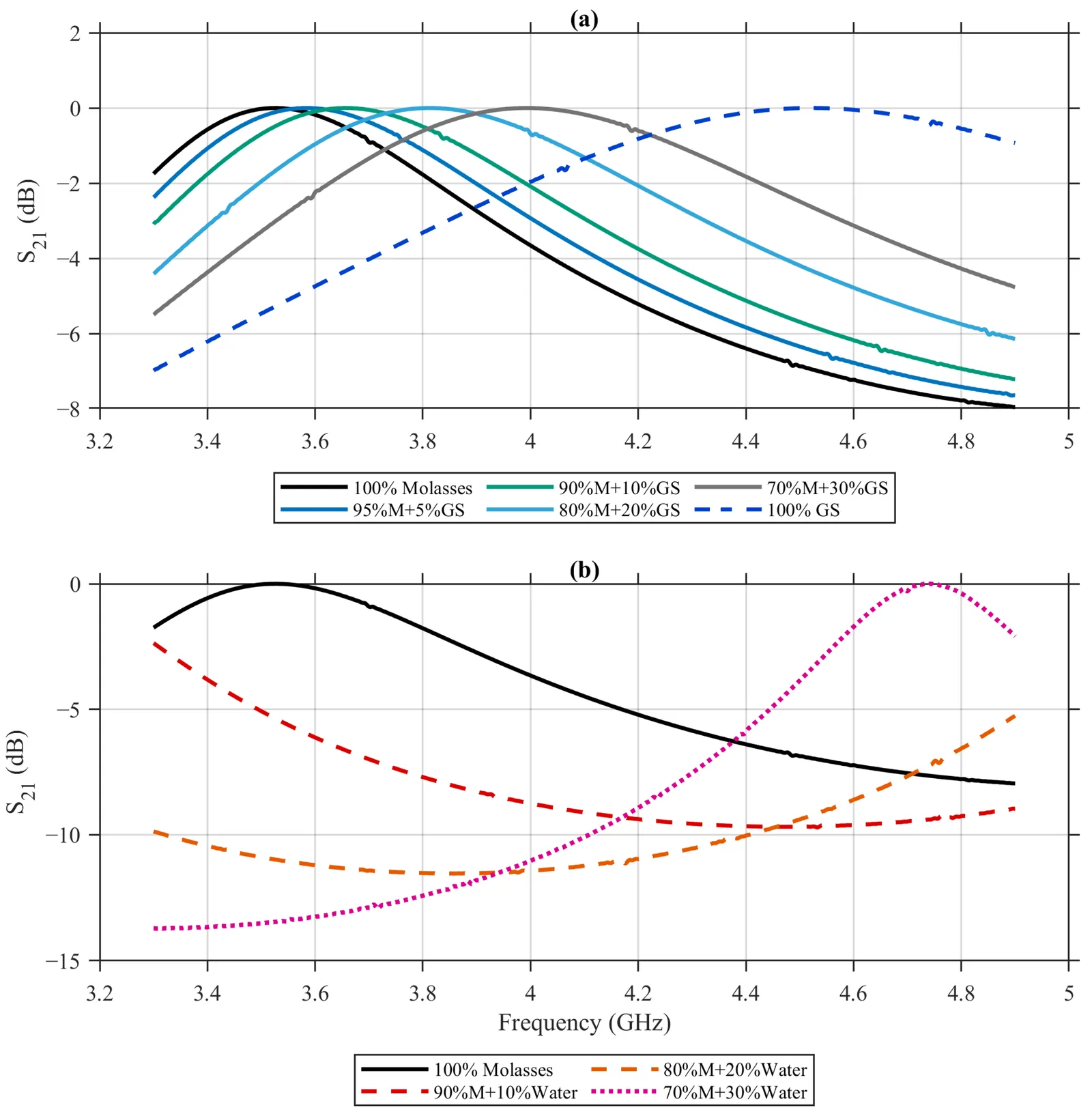
Mean S_21_ spectra of pure grape molasses, glucose syrup–adulterated mixtures, pure glucose syrup, and water-adulterated mixtures over the 3.30–4.90 GHz frequency range. Each curve represents the point-wise mean of five consecutive technical measurements. (**a**) Grape molasses-Glucose syrup mixtures; (**b**) Grape molasses-Water mixtures.

The S_21_ spectra provided complementary information, especially for water adulteration. Pure grape molasses had a mean S_21_ value of −4.1199 ± 0.0001 dB, whereas pure glucose syrup had a mean S_21_ value of −2.2371 ± 0.0002 dB. The glucose syrup mixtures showed moderate transmission loss, with mean S_21_ values ranging from −3.7352 ± 0.0002 dB for the 5% glucose syrup mixture to −2.1536 ± 0.0003 dB for the 30% glucose syrup mixture. In contrast, the water-diluted mixtures exhibited stronger attenuation, with mean S_21_ values of −8.0168 ± 0.0002 dB, −10.0712 ± 0.0002 dB, and −8.4294 ± 0.0002 dB for the 10%, 20%, and 30% water mixtures, respectively. These results indicate that S_11_ mainly captured resonance displacement associated with glucose syrup substitution, whereas S_21_ more clearly reflected transmission loss associated with water dilution. The mean S_21_ spectra are shown in Figure 5, and the group-wise mean S_21_ values are summarized in Table 2.

The five consecutive technical measurements showed low short-term variation for all mixture groups. The mean point-wise standard deviations (SD) of both S_11_ and S_21_ were approximately 0.006–0.007 dB, indicating good repeatability under the same fixture-loading condition. The S_11_ resonance frequency was also highly repeatable for most groups. Among the water-diluted samples, the 70M30W sample showed a resonance-like minimum near the upper boundary of the measured frequency range. Therefore, the resonance frequency of this water-diluted sample should be interpreted cautiously. The repeatability statistics of the main S-parameter features extracted from the five consecutive technical measurements are summarized in Table 3.

### 3.2. Regression Analysis for Glucose Syrup Estimation

The relationship between S_11_ resonance frequency and glucose syrup content was evaluated using linear regression. This analysis was performed to determine whether the S_11_ resonance frequency could be used as a simple quantitative indicator of glucose syrup substitution under controlled mixture conditions. Two calibration ranges were considered. The first model was fitted over the practical adulteration range of 0–30% glucose syrup, including pure grape molasses as the 0% reference. This range represents the main adulteration interval evaluated in the study. The second model included pure glucose syrup as the 100% endpoint reference. This model was used to describe the broader endpoint-supported calibration trend. In the practical 0–30% range, S_11_ resonance frequency increased almost linearly with glucose syrup content. The relationship between S_11_ resonance frequency and glucose syrup content for this range is given in Equation (1):

(1) $$f_{res}\;=\;0.015655\;\times \;GS\%\;+\;3.5109$$

where $f_{res}$ is the S_11_ resonance frequency in GHz and GS% is the glucose syrup content. This model produced a calibration R^2^ = 0.9955, RMSE_fres_ = 0.0113 GHz, and a calibration-based inverse prediction error of 0.72 percentage points; these values were computed on the same mixtures used to fit the regression and do not represent independent external prediction performance. These results indicate that S_11_ resonance frequency was highly sensitive to glucose syrup substitution within the practical adulteration range.

When pure glucose syrup was included as the 100% endpoint reference, the broader endpoint-supported relationship is given in Equation (2):

(2) $$f_{res}\;=\;0.009824\times GS\%+3.5789$$

The endpoint-supported model produced R^2^ = 0.9677, RMSE$f_{res}$ = 0.0608 GHz, and an inverse prediction error of 6.19 percentage points. Although this model describes the broader electromagnetic trend from pure grape molasses to pure glucose syrup, it should not be interpreted as a general commercial prediction model. The 100% glucose syrup group represents an endpoint reference rather than a realistic adulteration level.

**Figure 6:**
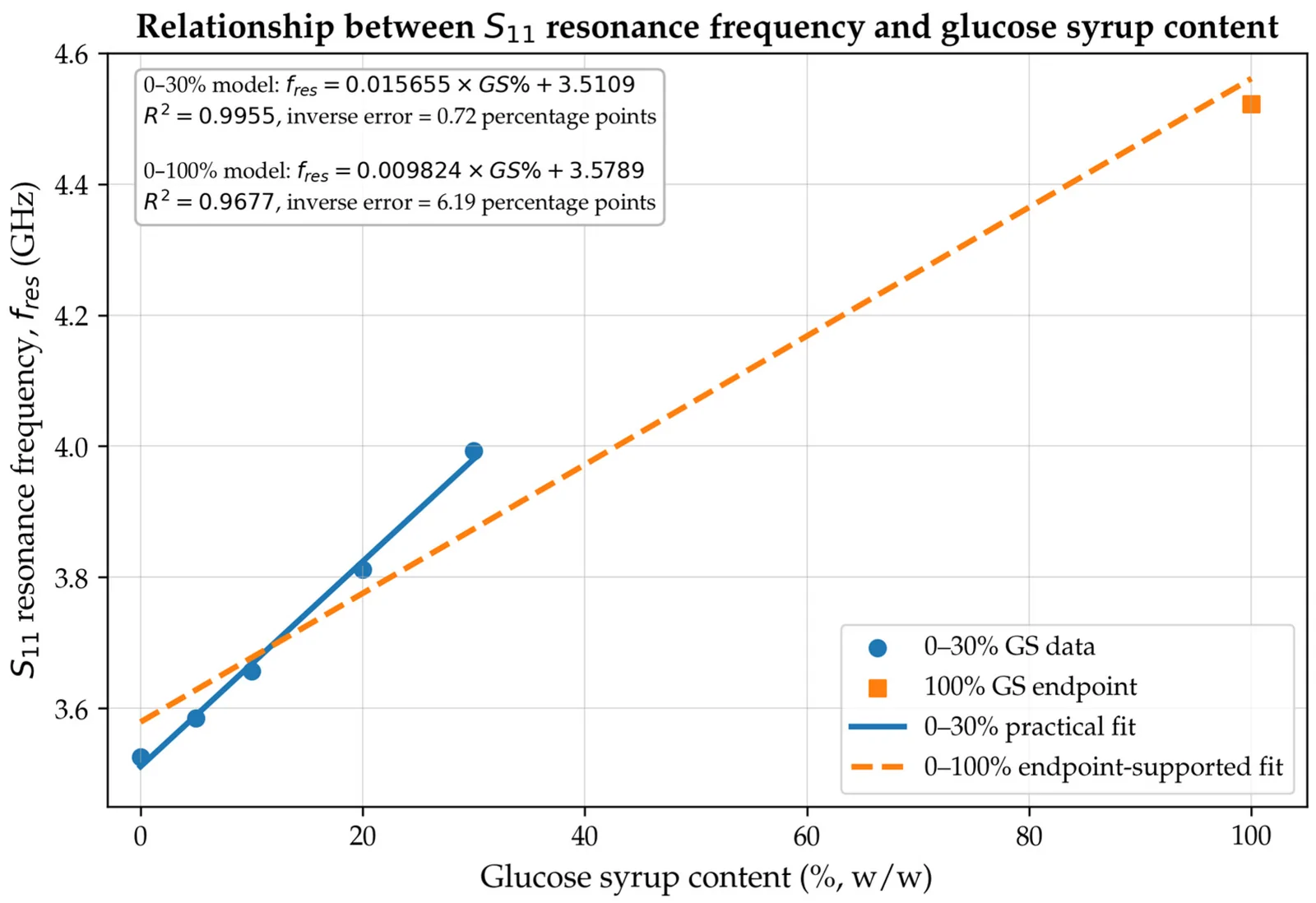
Relationship between S_11_ resonance frequency and glucose syrup content. The practical adulteration model was fitted using the 0–30% glucose syrup range, whereas the endpoint-supported model included pure glucose syrup as the 100% reference.

Overall, the regression results show that S_11_ resonance frequency can serve as a physically interpretable indicator of glucose syrup substitution. The practical 0–30% model is more relevant for adulteration screening, whereas the 0–100% model provides an endpoint-supported calibration trend. The regression results are shown in Figure 6 and summarized in Table 4.

### 3.3. Classification Analysis of Adulteration Mechanisms

Group-blocked point-wise classification was performed to evaluate whether the combined frequency, S_11_, and S_21_ responses could distinguish glucose syrup substitution from water dilution. The pure grape molasses group was excluded from the main binary task. The analysis included five glucose syrup-related group-mean spectra and three water-diluted group-mean spectra. Although these spectra contained 8008 frequency-wise observations in total, these observations were nested within only eight physical mixture groups and were not statistically independent samples. Therefore, the effective independent sample size for the classification analysis was eight mixture groups. Leave-one-mixture-group-out validation was used so that all frequency points from the same physical mixture group were held out together during testing.

**Figure 7:**
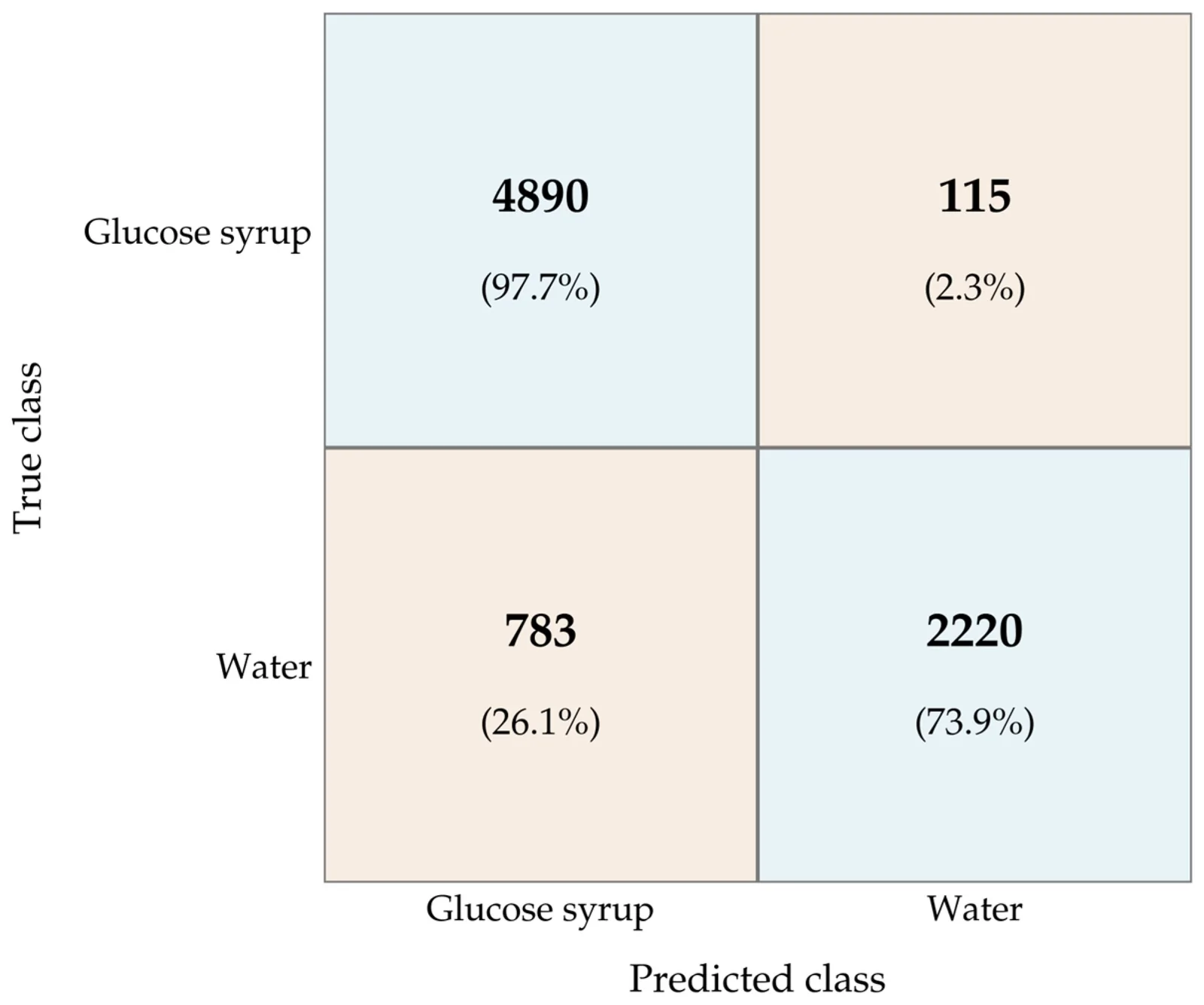
Confusion matrix of the best-performing group-blocked point-wise classifier for distinguishing glucose syrup substitution from water dilution. Percentages indicate pooled row-wise classification rates across leave-one-mixture-group-out folds. The displayed frequency-wise predictions are nested within eight physical mixture groups and therefore should not be interpreted as independent sample counts.

The comparison of ten classifiers is presented in Table 5. Among the evaluated models, the Ensemble Bagged Trees (EBT) classifier achieved the highest performance, with an accuracy of 88.79%, balanced accuracy of 85.81%, and macro-F1 score of 0.874. Linear SVM showed the second-best performance, with an accuracy of 82.53% and a macro-F1 score of 0.811. Logistic Regression showed a similar result, with an accuracy of 82.31% and a macro-F1 score of 0.809. Radial basis function support vector machine (RBF-SVM) also performed well, reaching 81.06% accuracy and a macro-F1 score of 0.799. In contrast, k-nearest neighbors (KNN) showed the lowest performance under the same group-blocked validation strategy. For the best-performing EBT model, the pooled point-wise recall was 0.977 for the glucose syrup class and 0.739 for the water class. These values were calculated from frequency-wise predictions pooled across the leave-one-mixture-group-out folds and should not be interpreted as performance estimates from thousands of independent samples. The confusion matrix of the EBT model is shown in Figure 7 for descriptive visualization of the classification pattern. The model separated glucose syrup substitution more successfully than water dilution, suggesting that some frequency regions of the water-diluted spectra overlapped with the glucose syrup class. Because the effective independent sample size was only eight physical mixture groups, these results should be interpreted as exploratory evidence of spectral separability rather than as statistically precise estimates of diagnostic performance. The model separated glucose syrup substitution more successfully than water dilution, suggesting that some water-diluted observations partially overlapped with the glucose syrup class in specific frequency regions. Because only one physical mixture group was held out in each fold and the study included only eight independent mixture groups, robust group-level uncertainty estimates could not be derived from the present dataset without overstating the available statistical information. Accordingly, the pooled classification metrics are reported only as exploratory measures of spectral separability. Therefore, the classification results should be interpreted as leakage-controlled proof-of-concept evidence of separability rather than as final diagnostic performance.

The main experimental findings are summarized in Table 6. Overall, glucose syrup substitution and water dilution produced different microwave S-parameter responses. S_11_ resonance frequency mainly reflected glucose syrup substitution, whereas S_21_ transmission loss was more informative for water dilution. The group-blocked classification results further showed that the combined frequency, S_11_, and S_21_ features provided leakage-controlled evidence for distinguishing the two adulteration mechanisms, with Ensemble Bagged Trees giving the highest performance among the evaluated classifiers.

Overall, the results demonstrate that glucose syrup substitution and water dilution produced distinct microwave S-parameter responses. Glucose syrup substitution was mainly associated with a systematic shift in S_11_ resonance frequency, whereas water dilution was more clearly reflected by increased S_21_ transmission loss. The repeatability statistics, calibration results, and group-blocked classification findings provide preliminary support for the feasibility of the proposed approach as a proof-of-concept screening method under the controlled conditions of this study. However, the classification performance should be interpreted as leakage-controlled evidence of separability rather than as a fully validated diagnostic result.

## 4. Discussion

This study investigated whether broadband microwave S-parameter measurements could provide physically interpretable indicators for detecting glucose syrup substitution and water dilution in grape molasses. The results showed that these two adulteration mechanisms produced different electromagnetic responses. Glucose syrup substitution mainly affected the S_11_ resonance frequency, whereas water dilution was more clearly reflected by increased S_21_ transmission loss. However, complex permittivity, dielectric loss, electrical conductivity, moisture content, soluble solids, and viscosity were not measured in the present study. Therefore, the observed S_11_ resonance shifts and S_21_ attenuation changes should be interpreted as physically plausible S-parameter indicators rather than quantitatively confirmed dielectric mechanisms. The most prominent response in the glucose syrup series was the systematic upward shift in S_11_ resonance frequency. This behavior may be associated with changes in the effective dielectric properties of the sample-loaded waveguide section. Grape molasses and glucose syrup differ in sugar composition, water activity, ionic content, viscosity, and molecular mobility. These compositional differences can influence the effective permittivity and loss characteristics of the mixture. From a simple size-resonance perspective, the resonance frequency of the sample-loaded region depends on the effective permittivity of the liquid mixture. This qualitative interpretation is consistent with published dielectric measurements of water, grapes, sugar solutions, and glucose solutions. Water exhibits a comparatively high dielectric permittivity, although its complex permittivity decreases markedly with increasing microwave frequency and depends strongly on temperature [26]. Tulasidas et al. [27] experimentally showed at 2.45 GHz that the dielectric constant and loss factor of grapes and sugar solutions vary substantially with moisture content, sugar concentration, and temperature. Smulders et al. [28] likewise demonstrated frequency- and concentration-dependent complex permittivity for aqueous glucose solutions over 0.5–67 GHz. These literature data support the expectation that replacing part of a sugar-rich food matrix or adding water can alter the effective dielectric loading of the microwave fixture. However, published values from grapes or aqueous sugar/glucose solutions cannot be directly assigned to the commercial grape molasses and glucose syrup used in the present experiment. Therefore, the observed upward resonance shift may be associated with changes in the effective dielectric properties of the mixture as glucose syrup replaces grape molasses. However, because the complex permittivity of the grape molasses and glucose syrup used in this study was not directly measured, neither the magnitude nor the direction of the sample-specific permittivity change can be experimentally confirmed from the present data. Accordingly, this interpretation should be regarded as a literature-supported qualitative hypothesis rather than a verified dielectric mechanism. Similarly, water addition is expected to modify the dielectric response of the mixture because water has a comparatively high microwave permittivity and loss contribution. However, since the complex permittivity and dielectric loss of the present water-diluted samples were not directly measured, the observed S_11_ and S_21_ changes cannot be quantitatively attributed to a specific permittivity or loss variation. The water-related interpretation should therefore also be regarded as literature-supported and qualitative. Under controlled mixture conditions, S_11_ resonance frequency therefore appears to be a simple and physically interpretable indicator of glucose syrup substitution.

The regression results further support this interpretation. The practical 0–30% glucose syrup model showed a very strong linear relationship between S_11_ resonance frequency and glucose syrup content, indicating high sensitivity within the adulteration range evaluated in this study. The endpoint-supported 0–100% model also showed a strong relationship; however, its prediction error was higher because the pure glucose syrup group represented an electromagnetic endpoint rather than a realistic commercial adulteration level. For this reason, the 0–30% model is more relevant for practical adulteration screening, whereas the 0–100% model should be interpreted as a broader endpoint-supported calibration trend. Because resonance frequency is related to the square root of the effective permittivity, the linear 0–30% trend is not expected to fully describe the 100% glucose syrup endpoint. Water dilution produced a different microwave response. Unlike glucose syrup substitution, water addition did not generate a consistent and easily quantifiable S_11_ resonance shift across all water-diluted samples. Instead, the water-diluted mixtures showed stronger S_21_ attenuation. This result is physically reasonable because S_21_ is directly related to transmission loss and dielectric absorption. The stronger attenuation observed in the water-diluted mixtures indicates that S_21_ is more informative than S_11_ for identifying water addition. The non-monotonic S_21_ behavior observed at higher water content may be associated with impedance mismatch, sample loading, or resonance-related effects, and should be verified using independently prepared samples. The repeatability results indicate that the WR-229 waveguide-based measurement setup produced stable short-term responses under the same fixture-loading condition. However, the five repeated measurements were technical repetitions, not independent sample batches. Therefore, they support measurement repeatability but do not increase the number of independent physical samples. This distinction is important because the main limitation of the present study is the limited number of physical mixture groups.

Machine learning was not intended to replace the physical interpretation of the S-parameter responses. It was used as an exploratory tool to evaluate whether the combined frequency, S_11_, and S_21_ patterns could separate the two adulteration mechanisms under a group-blocked validation strategy. The group-blocked classification results also support the presence of discriminative information in the combined S-parameter data. Among the ten evaluated classifiers, Ensemble Bagged Trees achieved the best performance in distinguishing glucose syrup substitution from water dilution, with 88.79% accuracy and a macro-F1 score of 0.874. Linear SVM, Logistic Regression, and RBF-SVM also produced comparatively strong results, indicating that the separation between the two adulteration mechanisms was not limited to a single model family. The higher performance of the ensemble bagging approach suggests that combining multiple decision trees can better capture the local decision structure of the frequency, S_11_, and S_21_ responses while retaining a leakage-controlled evaluation design. Nevertheless, the classification model should not be interpreted as a finalized diagnostic classifier. Because all frequency points belonging to the same physical mixture group were held out together during validation, the reported performance provides a leakage-controlled estimate of separability. However, the number of independent physical mixture groups was limited. Therefore, the classification results should be considered exploratory evidence that the combined S_11_ and S_21_ responses contain useful information for separating the two adulteration mechanisms.

The present findings are consistent with previous studies showing that spectroscopic, dielectric, and microwave-based measurements can provide useful information for food adulteration screening. Yaman and Durakli Velioglu [9] demonstrated that ATR-FTIR spectroscopy combined with chemometric methods can be used for detecting glucose syrup adulteration in grape, carob, and mulberry pekmez. Naderi-Boldaji et al. [13] also showed that dielectric spectroscopy combined with multivariate classification methods can identify adulteration in grape syrup. In microwave-based applications, Hasar et al. [14] used S_21_-based measurements to quantify water adulteration in honey, while recent studies have combined microwave S-parameter measurements with machine learning for food authentication in other matrices [16,17]. Compared with these studies, the present work focuses specifically on grape molasses and evaluates the complementary roles of S_11_ resonance frequency and S_21_ transmission loss under a leakage-controlled group-blocked validation strategy. Compared with confirmatory methods such as stable isotope ratio analysis or ATR-FTIR chemometric models, microwave S-parameter sensing should be regarded as a rapid screening approach rather than a replacement for laboratory-based authentication. Its potential advantage lies in its rapid, non-destructive, and physically interpretable measurement principle. A practical screening strategy may use S_11_ resonance frequency as an indicator of glucose syrup substitution and S_21_ transmission loss as an indicator of water dilution. Samples flagged by such a screening method would still require confirmation by established analytical techniques.

This study has several limitations. Only nine physical mixture groups were prepared, and all mixtures were based on one commercial grape molasses sample. The glucose syrup and water adulteration levels were controlled, but independent batches, different grape molasses sources, and different syrup sources were not included. In addition, conventional physicochemical parameters such as Brix, pH, viscosity, density, moisture content, and electrical conductivity were not measured. Critically, complex permittivity and dielectric loss—the properties that most directly underlie the proposed resonance-shift interpretation—were also not measured on the present samples; the discussion of permittivity effects therefore relies on literature values for water and sugar solutions rather than on sample-specific dielectric characterization. The regression errors reported in this study were calculated from the calibration data and should therefore be interpreted as calibration performance under controlled mixture conditions. They do not represent independent external prediction performance. Although group-blocked validation reduced leakage between training and test groups, the classification dataset was still derived from only eight group-mean spectra. Therefore, the reported classification metrics should be interpreted as exploratory evidence of separability rather than as proof of generalizable diagnostic performance.

The present revision does not resolve the limitation of independent sample preparation or external validation, because no additional physical mixture batches were available. Accordingly, the current regression and classification results should be regarded as within-study pilot evidence rather than independently validated predictive performance. Future studies should include multi-batch samples, additional adulterants such as fructose syrup, sucrose syrup, and sugar beet syrup, repeated measurements across different days, temperature-controlled experiments, dielectric characterization of the samples (e.g., via open-ended coaxial probe measurements), and conventional physicochemical analyses. These improvements are necessary before the proposed microwave approach can be considered for practical deployment. Despite these limitations, the present results provide a coherent proof-of-concept framework: S_11_ resonance frequency provides a simple indicator for glucose syrup substitution, S_21_ transmission loss provides complementary information for water dilution, and leakage-controlled classification provides exploratory evidence that the combined S-parameter features contain discriminative information for separating these two adulteration mechanisms under the controlled conditions evaluated.

## 5. Conclusions

This study provides proof-of-concept evidence that broadband microwave S-parameter measurements may offer useful indicators for screening glucose syrup substitution and water dilution in grape molasses under controlled single-source conditions. The two adulteration mechanisms produced different electromagnetic responses in the 3.30–4.90 GHz frequency range. The detection principle relies on the complementary behavior of the two S-parameters: S_11_ resonance frequency reflects resonance changes in the sample-loaded waveguide section, whereas S_21_ transmission loss reflects attenuation associated with the liquid mixture. Glucose syrup substitution was mainly associated with a systematic upward shift in S_11_ resonance frequency. In the practical 0–30% adulteration range, S_11_ resonance frequency showed a very strong linear relationship with glucose syrup content on the calibration data, with R^2^ = 0.9955 and a calibration-based inverse prediction error of 0.72 percentage points; these figures should not be read as independently validated prediction accuracy. The broader 0–100% endpoint-supported model also showed a strong relationship, but with higher prediction error. Therefore, the 0–30% model is more relevant for practical screening, whereas the 0–100% model should be interpreted as an endpoint-supported calibration trend. Water dilution produced a different response and was more clearly reflected by increased S_21_ transmission loss rather than by a consistent S_11_ resonance shift. These findings indicate that S_11_ and S_21_ provide complementary information: S_11_ resonance frequency is more suitable for glucose syrup substitution, while S_21_ transmission loss is more informative for water dilution. In the group-blocked glucose syrup versus water classification task, the Ensemble Bagged Trees classifier showed the highest pooled point-wise performance among the evaluated models, with an accuracy of 88.79% and a macro-F1 score of 0.874 across eight physical mixture groups. Because all frequency points belonging to the same physical mixture group were left out together during validation, this result represents a leakage-controlled estimate of separability rather than final diagnostic performance.

Overall, the proposed approach should be regarded as a rapid, non-destructive, and physically interpretable screening method, not as a replacement for confirmatory analytical techniques. Future studies should include real market samples, independently prepared simulated adulterated samples from different batches, different grape molasses and syrup sources, additional adulterants, temperature-controlled measurements, fully external validation sets, and conventional physicochemical analyses such as Brix, pH, viscosity, density, moisture content, and electrical conductivity. These steps are necessary to evaluate the generalizability and practical deployment potential of microwave S-parameter-based screening for grape molasses adulteration.

## Figures and Tables

**Table 1:** Sample groups, *w*/*w* mixture compositions, technical repetitions, and dataset structure.

Group ID	Composition	Adulterant Type	Technical Repetitions	Spectral Points per Repetition	Total Spectral Points Before Averaging
100M	100% grape molasses	Pure	5	1001	5005
95M5GS	95% grape molasses + 5% glucose syrup	Glucose syrup	5	1001	5005
90M10GS	90% grape molasses + 10% glucose syrup	Glucose syrup	5	1001	5005
80M20GS	80% grape molasses + 20% glucose syrup	Glucose syrup	5	1001	5005
70M30GS	70% grape molasses + 30% glucose syrup	Glucose syrup	5	1001	5005
100GS	100% glucose syrup	Endpoint reference	5	1001	5005
90M10W	90% grape molasses + 10% water	Water	5	1001	5005
80M20W	80% grape molasses + 20% water	Water	5	1001	5005
70M30W	70% grape molasses + 30% water	Water	5	1001	5005
Total					45,045

**Table 2:** Group-wise mean S_21_ values calculated from five consecutive technical measurements.

Group ID	Composition	Mean S_21_ ± SD (dB)
100M	100% grape molasses	−4.1199 ± 0.0001
95M5GS	95% grape molasses + 5% glucose syrup	−3.7352 ± 0.0002
90M10GS	90% grape molasses + 10% glucose syrup	−3.3225 ± 0.0001
80M20GS	80% grape molasses + 20% glucose syrup	−2.6292 ± 0.0002
70M30GS	70% grape molasses + 30% glucose syrup	−2.1536 ± 0.0003
100GS	100% glucose syrup	−2.2371 ± 0.0002
90M10W	90% grape molasses + 10% water	−8.0168 ± 0.0002
80M20W	80% grape molasses + 20% water	−10.0712 ± 0.0002
70M30W	70% grape molasses + 30% water	−8.4294 ± 0.0002

**Table 3:** Repeatability statistics of the main S-parameter features extracted from five consecutive technical measurements.

Group ID	$f_{res}$ Mean ± SD (GHz)	S_11_min Mean ± SD (dB)	S_21_min Mean ± SD (dB)	Mean Point-Wise SD S_11_ (dB)	Mean Point-Wise SD S_21_ (dB)
100M	3.5256 ± 0.0000	−83.2432 ± 0.0111	−7.9618 ± 0.0073	0.0066	0.0064
95M5GS	3.5848 ± 0.0000	−56.3958 ± 0.0036	−7.6553 ± 0.0053	0.0065	0.0065
90M10GS	3.6568 ± 0.0000	−64.8794 ± 0.0023	−7.2202 ± 0.0028	0.0064	0.0064
80M20GS	3.8120 ± 0.0000	−60.8461 ± 0.0049	−6.1520 ± 0.0053	0.0064	0.0067
70M30GS	3.9928 ± 0.0000	−63.6396 ± 0.0089	−5.4986 ± 0.0066	0.0066	0.0065
100GS	4.5224 ± 0.0000	−62.3309 ± 0.0021	−6.9843 ± 0.0083	0.0062	0.0064
90M10W	3.3000 ± 0.0000	−3.7671 ± 0.0060	−9.7125 ± 0.0116	0.0065	0.0065
80M20W	4.8997 ± 0.0007	−1.5374 ± 0.0027	−11.5497 ± 0.0054	0.0065	0.0065
70M30W	4.7384 ± 0.0000	−52.4609 ± 0.0050	−13.7291 ± 0.0058	0.0062	0.0065

**Table 4:** Linear regression calibration results for glucose syrup content (computed on calibration data; not independently validated) estimation using S_11_ resonance frequency.

Calibration Range	R^2^	RMSE (GHz)	Inverse Prediction Error
0–30% GS	0.9955	0.0113	0.72 percentage points
0–100% GS	0.9677	0.0608	6.19 percentage points

**Table 5:** Group-blocked classification performance for distinguishing glucose syrup substitution from water dilution using group-mean S-parameter spectra.

Model	Accuracy (%)	Balanced Accuracy (%)	Macro Precision	Macro Recall	Macro-F1
Ensemble Bagged Trees	88.79	85.81	0.906	0.858	0.874
Linear SVM	82.53	80.78	0.816	0.808	0.811
Logistic Regression	82.31	80.52	0.813	0.805	0.809
RBF-SVM	81.06	79.98	0.798	0.800	0.799
Gaussian Naive Bayes	79.51	80.30	0.786	0.803	0.789
Gradient Boosting	76.64	75.89	0.752	0.759	0.755
Decision Tree	73.50	73.38	0.723	0.734	0.725
Random Forest	72.58	72.97	0.717	0.730	0.718
Extra Trees	72.10	72.46	0.712	0.725	0.713
KNN	70.05	69.64	0.687	0.696	0.689

**Table 6:** Summary of main experimental findings.

Finding	Result	Interpretation
Dataset structure	9 physical mixture groups were measured. 5 technical repetitions were recorded for each group, producing 45 measured spectra and 45,045 spectral points before averaging. Classification was performed using group-mean spectra.	Technical repetitions improved repeatability assessment but were not treated as independent physical samples.
S_11_ behavior	S_11_ resonance frequency shifted from 3.5256 GHz in pure grape molasses to 4.5224 GHz in pure glucose syrup.	S_11_ resonance frequency was sensitive to glucose syrup substitution.
S_21_ behavior	Water mixtures showed stronger transmission loss, with mean S_21_ values of −8.0168, −10.0712, and −8.4294 dB for 10%, 20%, and 30% water, respectively.	S_21_ was more informative for water dilution.
Regression performance	In the practical 0–30% glucose syrup range, S_11_ resonance frequency showed R^2^ = 0.9955 and a calibration-based inverse prediction error of 0.72 percentage points; these values were not independently validated.	S_11_ resonance frequency provided a simple calibration-based quantitative indicator of glucose syrup substitution under controlled conditions.
Classification performance	In group-blocked glucose syrup versus water classification, Ensemble Bagged Trees achieved a pooled point-wise accuracy of 88.79% and a macro-F1 score of 0.874 across eight physical mixture groups.	Frequency, S_11_, and S_21_ together provided leakage-controlled exploratory evidence for separating the two adulteration mechanisms; the effective independent sample size was eight physical mixture groups.
Main limitation	The classification analysis was based on a limited number of physical mixture groups and group-mean spectra.	The results should be interpreted as screening-level evidence rather than final diagnostic performance.

## Data Availability

The data supporting the findings of this study are available from the corresponding author upon reasonable request.
